# Cholecystectomy in a patient with paroxysmal nocturnal haemoglobinuria undergoing ravulizumab maintenance treatment

**DOI:** 10.1002/jha2.336

**Published:** 2021-11-09

**Authors:** Mitsuru Moriyama, Yasuo Aota, Masahiro Okabe, Yoshiaki Osaka, Seiichiro Katagiri, Daigo Akahane, Akihiko Gotoh

**Affiliations:** ^1^ Department of Hematology Tokyo Medical University Tokyo Japan; ^2^ Department of Internal Medicine Kohsei Chuo General Hospital Tokyo Japan; ^3^ Deptartment of Gastrointestinal and General Surgery Kohsei Chuo General Hospital Tokyo Japan

**Keywords:** cholecystectomy, paroxysmal nocturnal haemoglobinuria, ravulizumab, tumour necrosis factor‐α

## Abstract

A 47‐year‐old male with paroxysmal nocturnal haemoglobinuria (PNH) controlled with routine ravulizumab administration suffered a massive haemolytic crisis due to choledocholithiasis. Laparoscopic cholecystectomy was performed 6 weeks after a regular ravulizumab infusion. After surgery, the patient presented with anaemia without marked elevation in lactate dehydrogenase and required two blood transfusions. Tumour necrosis factor‐*α* increased more than twofold with reticulocyte suppression after surgery, suggesting the involvement of myelosuppressive cytokines. This case suggests that laparoscopic surgery may be safely performed in patients with PNH receiving ravulizumab maintenance treatment. However, attention should be paid to postoperative anaemia, regardless of breakthrough haemolysis.

## 1 INTRODUCTION

Paroxysmal nocturnal haemoglobinuria (PNH) is a disease of chronic, uncontrolled, terminal complement activity resulting in intravascular haemolysis (IVH) and thrombosis. Complement protein 5 (C5) inhibitors, eculizumab and ravulizumab, are the current standard of care for patients with PNH. Eculizumab, the first humanised monoclonal antibody, is effective in patients with PNH [1]. However, approximately 27% of patients experience breakthrough haemolysis (BTH) due to insufficient complement suppression or complement activating conditions such as surgery[2]. Ravulizumab, the first long‐acting C5 inhibitor, provides immediate, complete, and sustained C5 inhibition for 8 weeks and prevents IVH and thrombosis.^2–5^ Surgery can exacerbate complement activation[6] and trigger severe IVH and thrombosis in patients with PNH during the perioperative period. However, ravulizumab may reduce the risk of BTH before or after surgery.^2,3^ Thus, ravulizumab is expected to contribute to better surgical outcomes. To our knowledge, detailed reports of patients with PNH who underwent surgery during ravulizumab treatment have yet to be described. We report a case of successful laparoscopic cholecystectomy during ravulizumab treatment in a patient with aplastic anaemia (AA)‐PNH.

## 2 CASE

A 47‐year‐old man diagnosed with AA‐PNH at the age of 15 years required regular red blood cell (RBC) transfusions, despite immunosuppressive treatment. Additionally, he presented with repeated IVH and cholecystitis episodes, which led to haemoglobinuria and severe anaemia. After the initiation of eculizumab treatment in December 2008, he became transfusion independent. Ravulizumab, which can be administered at 8‐week intervals, was approved in Japan in 2019, and a treatment switch from eculizumab to ravulizumab was made in December 2019 based on the patient's preference to reduce the number of hospital visits. Ravulizumab treatment decreased the frequency of haemolytic attacks and hospital visits and improved the patient's quality of life; further, his condition had remained stable.

In April 2020, he presented 10 days of continuous fever and general jaundice, for which he sought medical care. Laboratory test results showed elevated total bilirubin (27.86 mg/dl), lactate dehydrogenase (LDH 390 U/l), serum creatinine (1.56 mg/dl), C‐reactive protein (CRP 16.69 mg/dl), and reticulocytes (12.14%), and decreased haemoglobin (5.4 g/dl). The patient required RBC transfusions for three consecutive days to maintain haemoglobin above 6 g/dl. A computed tomography scan revealed inflammation of his bile duct. He was diagnosed with choledocholithiasis and had suffered a massive haemolytic crisis. After receiving antibiotics and hydration, the patient underwent endoscopic retrograde cholangiopancreatography, bile duct catheterisation, and stent placement. Thus, CRP and LDH returned to the normal range, and blood transfusion became unnecessary. The laparoscopic cholecystectomy was then scheduled but had to be postponed because of the COVID‐19 pandemic. The surgery was finally performed 6 weeks after his most recent ravulizumab administration.

**FIGURE 1:**
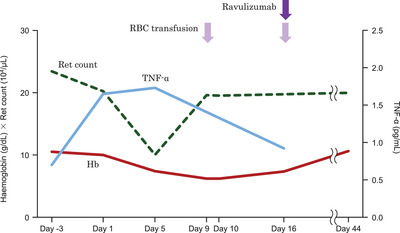
Changes in haemoglobin, reticulocytes, and tumour necrosis factor‐alpha (TNF‐*α*) during the pre‐ and postoperative periods. TNF‐*α* levels increased after the surgery, remained high until day 5, and reverted to near preoperative levels on day 16. In contrast, the reticulocyte count decreased up to day 5, and increased, thereafter, up to near preoperative levels (normal levels) on day 10. Abbreviations: Hb, haemoglobin; RBC, red blood cell; Ret, reticulocyte; TNF‐*α*, tumour necrosis factor‐alpha

The operating time was 3 h and 53 min, and the total blood loss volume was 275 ml. No RBC transfusions were required during the perioperative period. The postoperative proportion of PNH erythrocytes remained stable, with preoperative levels at 57.99%. The LDH was within the normal range (124–222 U/L), and the total bilirubin was not significantly elevated after the surgery (Table 1). No signs of apparent BTH were observed. However, the D‐dimer level slightly increased from 1.1 to 2.2 μg/ml after surgery (Table 1). He did not present any thromboembolic events pre‐ or postoperatively. The reticulocyte count was decreased on day 5 after the surgery but recovered on day 9 (Table 1; Figure 1). The 50% haemolytic complement (CH50) level showed a slight increase from below the measurement sensitivity from days 5–10 (Table 1), without obvious infection findings. LDH slightly exceeded the normal value on days 9 and 10, but returned to the normal range on day 16 (Table 1). The patient was discharged after a blood transfusion on day 10 after surgery. Ravulizumab was already scheduled to be routinely administered 16 days after surgery, and between days 10 and 16, the patient was resting at home to prevent unnecessary complement activation. At the outpatient visit 16 days after his surgery, his LDH level was determined to be within normal limits and no BTH was observed. However, his haemoglobin level was 7.3 g/dl with symptoms of anaemia, requiring another blood transfusion (Figure 1). On day 16, the regular ravulizumab dose was administered, after which no further blood transfusion was required, and haemoglobin returned to preoperative values (Figure 1).

**TABLE 1:** Laboratory parameters before and after the cholecystectomy

	**Preoperative**	**Postoperative**					
	**Day −3**	**Day 1**	**Day 5**	**Day 9**	**Day 10**	**Day 16**	**Day 44**
Haemoglobin (g/dl)	10.5	10.0	7.4	6.2	6.2	7.3	10.6
Reticulocyte count (10[4]/μl)	23.42	20.22	10.00	19.58	19.52	‐	‐
Total bilirubin (mg/dl)	4.60	5.10	2.90	3.11	3.14	3.00	3.17
Indirect bilirubin (mg/dl)	2.77	2.62	1.15	1.46	1.46	1.27	2.38
LDH (U/L; 124–222 normal)	192	202	215	223	223	209	181
CH50	<12.0	<12.0	24.1	21.0	22.0	‐	<8.0
CRP (mg/dl)	0.05	4.10	4.74	2.34	2.34	0.42	0.11
D‐dimer (μg/ml)	0.5	1.1	2.2	2.4	2.4	‐	0.5
CD59‐negative RBC (%)	59.97	54.11	57.99	‐	‐	‐	‐

Abbreviations: CH50, 50% haemolytic complement; CRP, C‐reactive protein; Day 1, day of the cholecystectomy; LDH, lactate dehydrogenase; RBC, red blood cell.

## 3 DISCUSSION

Patients with PNH are at a high risk of complement‐mediated IVH and thrombosis during or after surgery. Successful cases of cardiopulmonary bypass surgery and cholecystectomy have been reported in patients with PNH during eculizumab treatment [7, 8, 9, 10, 11]. These reports suggest that C5 inhibitor treatment prevents surgery‐induced BTH and thrombosis in patients with PNH.

Complement activity demonstrated by the CH50 test is the cause of residual IVH activity in patients receiving C5 inhibitor treatment [12]. In this case, ravulizumab suppressed the onset of severe BTH; however, the elevated CH50 level from days 5 to 9 (Table 1) indicates that complement activation might not be completely inhibited. This patient's surgery was delayed because of the COVID‐19 pandemic. Thus, the unplanned and prolonged interval from the previous ravulizumab administration might have impacted this result. We further analysed circulating tumour necrosis factor‐α (TNF‐α) and interferon‐γ (IFN‐γ) levels[13] in the patient's serum to determine possible causes of postoperative anaemia. Although IFN‐γ remained below the sensitivity level (data not shown), TNF‐*α* levels increased twofold after the surgery (possibly because of surgical inflammation) and then reverted to near preoperative levels (Figure 1). As one of the actions of TNF‐*α* is to suppress erythropoiesis, TNF‐*α* might have induced postoperative anaemia in our patient. Given the haemolytic and bone marrow failure aspects of PNH, greater sensitivity to inflammatory cytokines such as TNF‐α may contribute to the development of postoperative anaemia.

This case suggests that patients with PNH receiving ravulizumab treatment might be able to safely undergo surgery, even in cases in which surgery is performed 6 weeks after the previous ravulizumab infusion. Our case also suggests that minimally invasive surgeries, such as laparoscopic cholecystectomy, may not pose a high risk of BTH among patients under C5 inhibitor treatments. Increased perioperative CH50 activity indicates that the complement activation was not entirely suppressed in our case. Conducting the surgical procedure sooner than 6 weeks after the previous ravulizumab administration may further contribute to the prevention of perioperative complement activation. Furthermore, in patients with PNH, which is associated with impaired bone marrow function, surgical inflammation and cytokines released peri‐operatively could increase myelosuppression. Thus, careful observation of postoperative anaemia progression in patients with PNH is needed, even if BTH is not observed.

## CONFLICT OF INTEREST

AG received scholarships from Eisai, Ono Pharmaceutical, Taiho Pharmaceutical, Takeda Pharmaceutical, Nippon Shinyaku, Chugai Pharmaceutical, MSD, Otsuka Pharmaceutical, Sumitomo Dainippon Pharma, Nippon Shinyaku, Bayer, Daiichi‐Sankyo, and Nihon Pharmaceutical, and received remuneration for time and effort spent by the researcher for meeting attendance from Novartis Pharma, Alexion Pharma, Eisai, Ono Pharmaceutical, Taiho Pharmaceutical, Takeda Pharmaceutical, Nippon Shinyaku, Chugai Pharmaceutical, Otsuka Pharmaceutical, Sumitomo Dainippon Pharma, Daiichi‐Sankyo, Nihon Pharmaceutical, Kyowa Kirin, Janssen Pharmaceutical, Pfizer, and Sanofi.

## CONFLICT OF INTEREST

The authors declare no conflict of interest.

## AUTHOR CONTRIBUTIONS

AG designed the study. Mitsuru Moriyama and Akihiko Gotoh wrote the manuscript. All authors contributed to the patient's care, data correction, and drafting and revision of the manuscript.
